# Supplementary material for: Explicit and implicit locomotor learning in individuals with chronic hemiparetic stroke
Source: bioRxiv. 2024 Jul 3:2024.02.04.578807. Originally published 2024 Feb 6. Preprint. [Version 3] doi: 10.1101/2024.02.04.578807 (PMC10871205; doi:10.1101/2024.02.04.578807)
Supplement: Supplement 1 [file NIHPP2024.02.04.578807v3-supplement-1.pdf]

bioRxiv preprint doi: https://doi.org/10.1101/2024.02.04.578807; this version posted July 3, 2024. The copyright holder for this preprint (which was not certified by peer review) is the author/funder, who has granted bioRxiv a license to display the preprint in perpetuity. It is made available under aCC-BY 4.0 International license.

# Supplemental material

Explicit and implicit locomotor learning in individuals with chronic hemiparetic stroke.

**Authors**: Jonathan M. Wood, Elizabeth Thompson, Henry Wright, Liam Festa, Susanne M. Morton, Darcy S. Reisman, and Hyosub E. Kim

## Supplemental Figures: Individual model fits

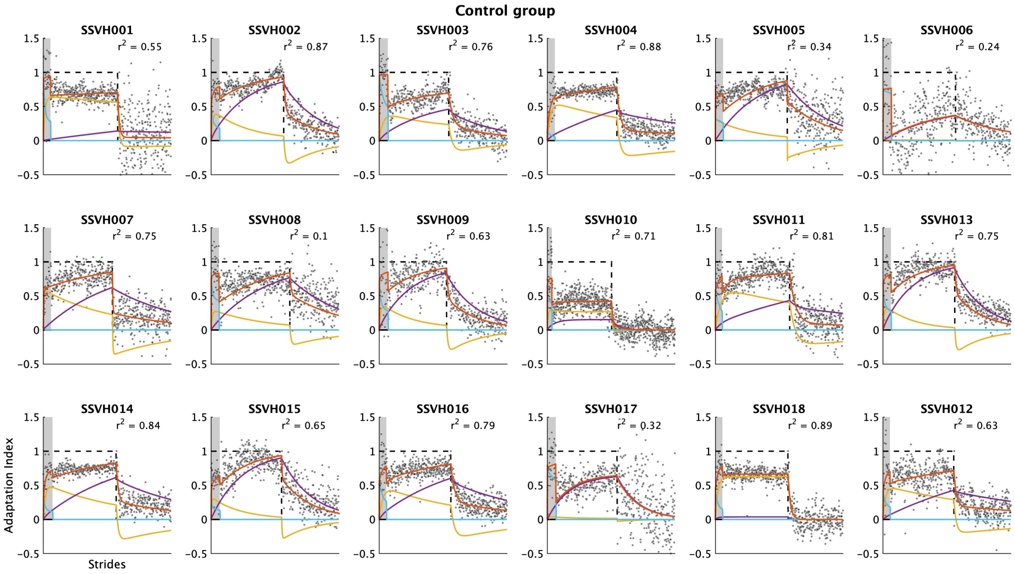


**Supplemental Figure S1 Individual model fits for the control group.** The voluntary correction model was fit to each participant's Adaptation Index data in the control group (n=18). Black dots represent the participant's Adaptation Index on that stride and the black dashed line represents the perturbation (which equals one during the Adaptation phase and 0 during De-adaptation phase). The red function represents the model's motor output (x), the light blue function (active only when the feedback is turned on – denoted by the gray shading) represents the explicit component ($x_{explicit}$), and the yellow and purple functions represent the fast ($x_{fast}$) and slow ($x_{slow}$) components of the implicit process, respectively.

bioRxiv preprint doi: https://doi.org/10.1101/2024.02.04.578807; this version posted July 3, 2024. The copyright holder for this preprint (which was not certified by peer review) is the author/funder, who has granted bioRxiv a license to display the preprint in perpetuity. It is made available under aCC-BY 4.0 International license.

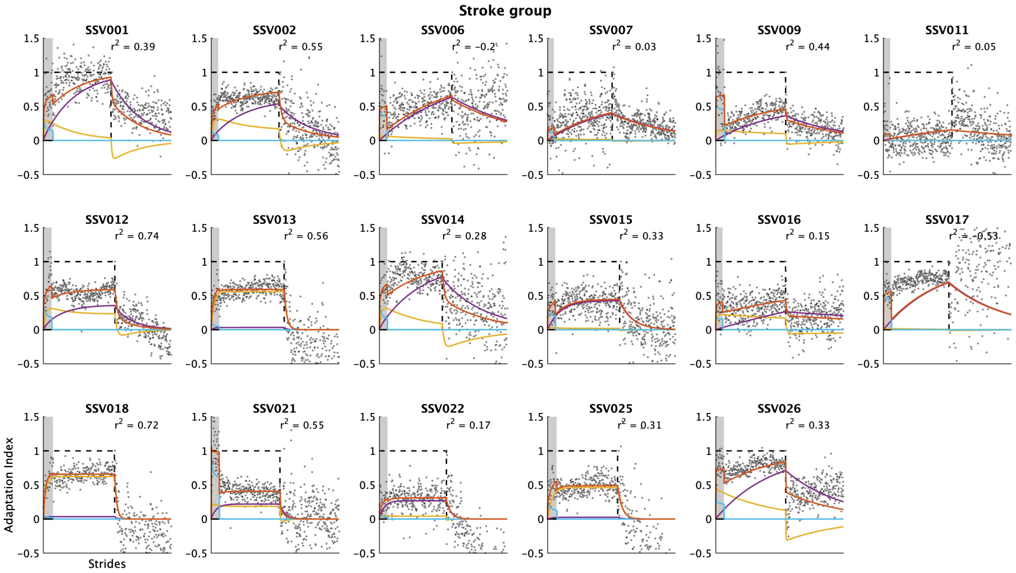


**Supplemental Figure S2 Individual model fits for the stroke group.** The voluntary correction model was fit to each participant's Adaptation Index data in the stroke group (n=17). Black dots represent the participant's Adaptation Index on that stride and the black dashed line represents the perturbation (which equals one during the Adaptation phase and 0 during De-adaptation phase). The red function represents the model's motor output (x), the light blue function (active only when the feedback is turned on – denoted by the gray shading) represents the explicit component ($x_{explicit}$), and the yellow and purple functions represent the fast ($x_{fast}$) and slow ($x_{slow}$) components of the implicit process, respectively.
