# Supplementary material for: Explicit and implicit locomotor learning in individuals with chronic hemiparetic stroke
Source: bioRxiv. 2024 Feb 6:2024.02.04.578807. Preprint. [Version 1] doi: 10.1101/2024.02.04.578807 (PMC10871205; doi:10.1101/2024.02.04.578807)
Supplement: Supplementary file 1 [file media-1.pdf]

Supplemental material

Explicit and implicit locomotor learning in individuals with chronic hemiparetic stroke.

Authors: Jonathan M. Wood, Elizabeth Thompson, Henry Wright, Liam Festa, Susanne M. Morton, Darcy S. Reisman, and Hyosub E. Kim

Supplemental methods

Data collection

During all phases of treadmill walking, individuals wore a ceiling mounted harness (that did not provide body weight support) and held a handrail to prevent falls. Additionally, we monitored heart rate using a Polar (Polar, Kempele, Finland) heart rate monitor for safety, and determined perceived exertion using the Borg Rate of Perceived Exertion (RPE) scale[1] after each walking phase. If participants exceeded >80% their age-predicted max heart rate, the treadmill was stopped, and the participant was provided with a seated rest break until their heart rate recovered. However, no included participants needed the treadmill to stop during any of the walking phases.

Participants walked on a dual belt treadmill that captured kinetic data through two force plates, one under each belt, at 1000 Hz (Bertec, Columbus, OH, USA). Kinematic data were captured and recorded at 100 Hz using a Vicon MX40, 8-camera motion capture system, and time-synchronized with the kinetic data in Nexus software (v2.8.2, Vicon Motion Systems, Inc., London, UK). We used a custom marker set with seven retroreflective markers, one for each heel, lateral malleolus, and fifth metatarsal head, and the left medial malleolus.

Data analysis

Step length, calculated in real-time using motion capture and the Motion Monitor, was defined as the anterior-posterior distance between the two ankle markers at heel strike. Heel strike was determined in real time using the following criteria: 1) The velocity of the heel markers changed from positive to negative; 2) a ground reaction force > one-third of the participants body weight detected through the treadmill force plate; and 3) no forces were detected through that same belt 4 ms prior to the detected step length.

The remainder of the data were analyzed with custom written MATLAB scripts (vR2022a, Mathworks, Natick, MA, USA). Step lengths were used to calculate step length asymmetry on each stride (s):

$$step\ length\ asymmetry_{[s]} = \frac{fast\ step\ length_{[s]} - slow\ step\ length_{[s]}}{fast\ step\ length_{[s]} + slow\ step\ length_{[s]}} * 100\% \quad (1)$$

Thus, values of 0 indicate perfect symmetry between the fast and slow step lengths, while values further from 0 indicate greater asymmetry. We baseline corrected this measure by subtracting the mean step length asymmetry during the Baseline phase from each stride in the experiment for each individual. We removed outlier step length asymmetry strides, defined as any step length asymmetry exceeding 3x the interquartile range of that participant's step length asymmetry (mean percent removed [min max] stroke = 0.14% [0.0 0.39]; control = 0.92% [0.0 2.32]). We also removed the first stride of each phase to account for treadmill acceleration.

We used step length asymmetry data to calculate an Adaptation Index for each individual for each stride (see equations 1a-c in the main text). During the Adaptation phase, an Adaptation index of 0 represents the minimum step length asymmetry (i.e., the max perturbation), and 1 indicates the perturbation has been fully corrected. During the De-adaptation phase, 1 is the maximum step length asymmetry (i.e., the max aftereffect) and 0 indicates the perturbation has been fully "washed-out" (i.e., baseline asymmetry has been restored).

Model fitting and model comparison

We fit the voluntary correction model to each participant's Adaptation Index data during the Adaptation and De-adaptation phases using MATLAB's fmincon function, setting the objective function as the sum of squared errors between the model output (x) and the data. All parameters were constrained between 0 and 1. Additionally, we constrained the fast-learning rate, B_{fast}, to be at least 5 times higher than the slow learning rate, B_{slow}; and the slow retention rate, A_{slow}, was constrained to be greater than the fast retention rate, A_{fast}.[2,3] To ensure stable fits, we initialized the implicit process parameters to the same values[2–4] based on a prior locomotor adaptation study in individuals with stroke[4]: $A_{fast} = 0.92$, $B_{fast} = 0.03$, $A_{slow} = 0.996$, $B_{slow} = 0.004$. The explicit parameter was initialized at uniformly random values between 0 and 1. To improve the stability of the $B_{explicit}$ parameter, we performed 10 initializations for each participant. We calculated model fits (r^2) using a bootstrapping procedure. We resampled with replacement the Adaptation and De-adaptation data 1000 times for each group, fitting the model to the bootstrapped samples. We report the mean and 95% confidence intervals of these r^2 values. We also calculated the r^2 of individual fits and report these the supplemental results, below.

To ensure that we did not overfit the data with the five-parameter, voluntary correction model, we also fit two simpler models to the data. For the single rate model, the motor output (x; i.e., Adaptation Index) is the result of a single process that has two parameters B, the learning rate, and A the retention rate[3,5]:

$$x_{[s+1]} = A\,x_{[s]} + B\,error_{[s]} \tag{2}$$

Additionally, we fit a four-parameter, dual rate model to the data.[2–4] This "dual-rate" model represents motor output (x; i.e., Adaptation Index) as the sum of a fast and slow process. This model is equivalent to the implicit process in the voluntary correction model:

$$x_{[s]} = x_{fast[s]} + x_{slow[s]} \tag{3}$$

$$x_{fast[s+1]} = A_{fast}x_{fast[s]} + B_{fast}error_{[s]} \tag{4}$$

$$x_{slow[s+1]} = A_{slow}x_{slow[s]} + B_{slow}error_{[s]} \tag{5}$$

Thus, neither alternative model includes a voluntary correction, or explicit learning component. We compared fits of the three different models using Akaike Information Criterion (AIC). We used a model recovery analysis to confirm that AIC was better than BIC at distinguishing between the three models for the current experiment.[6]

Statistical analysis

For our Bayesian statistical analysis, we assumed the Adaptation Index data at each timepoint of interest (Feedback On, Feedback Off, End Adaptation, Implicit Aftereffect) was sampled from a normal distribution, with a mean which depended on both within subject (i.e., time) and between subject (i.e., group) parameters as well as an interaction parameter. We confirmed a normal

distribution was a reasonable assumption for our data by observing favorable model comparison results against a student's t distribution. We calculated the posterior distribution for these means (and all the statistical model parameters) using Bayes rule, combining the evidence from our data with our prior assumptions about each parameter. The prior distributions for the between and within subject effects were set as a wide normal distribution centred on 0, and the prior for the standard deviation was set as a wide, positive-valued uniform distribution. Thus, our prior assumptions only served to make our inferences more conservative and did not bias the posterior. We calculated the joint posterior distribution in Python v4.3.0 using the PyMc 4 library[7] and the bayes-toolbox Python package.[8] We used Markov Chain Monte Carlo sampling to sample from joint posterior distribution 10,000 times with 2,000 tuning samples. We performed posterior predictive checks to ensure that the posterior samples accurately represented the data.[9,10]

Supplemental results

Individual model fits

Supplemental figures 1 and 2 display individual model fits with the individual r^2 value for that specific fit.

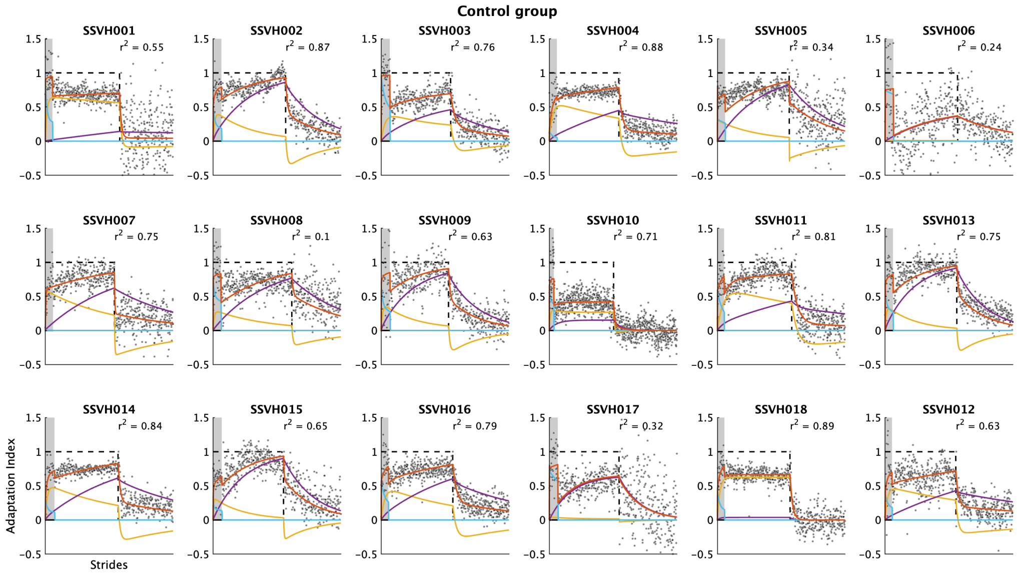

Supplemental Figure 1 Individual model fits for the control group. The voluntary correction model was fit to each participant's Adaptation Index data in the control group (n=18). Black dots represent the participant's Adaptation Index on that stride and the black dashed line represents the perturbation (which equals one during the Adaptation phase and 0 during De-adaptation phase). The red function represents the model's motor output (x), the light blue function (active only when the feedback is turned on – denoted by the gray shading) represents the explicit component ($x_{explicit}$), and the yellow and purple functions represent the fast (x_{fast}) and slow (x_{slow}) components of the implicit process, respectively.

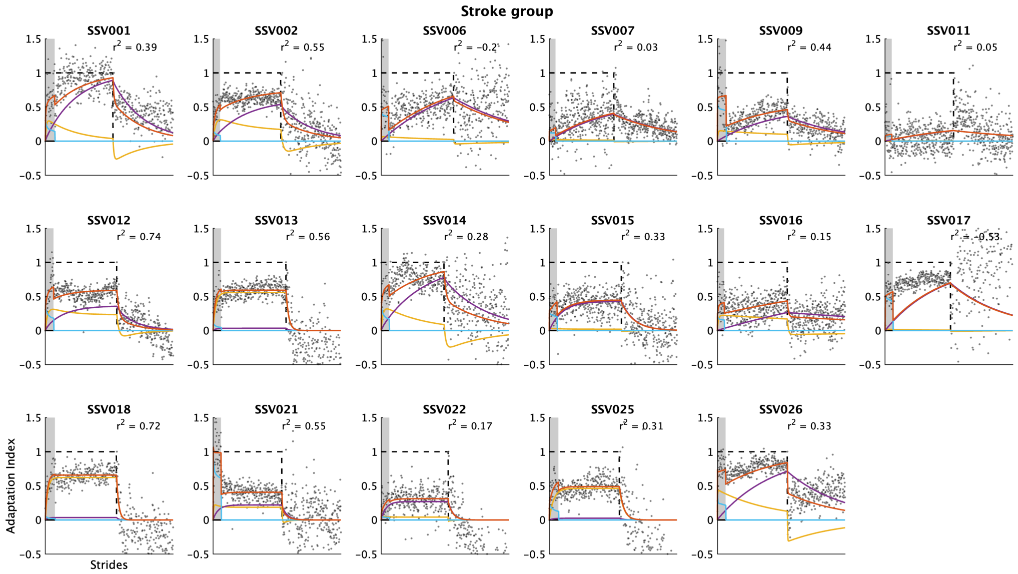

Supplemental Figure 2 Individual model fits for the stroke group. The voluntary correction model was fit to each participant's Adaptation Index data in the stroke group (n=17). Black dots represent the participant's Adaptation Index on that stride and the black dashed line represents the perturbation (which equals one during the Adaptation phase and 0 during De-adaptation phase). The red function represents the model's motor output (x), the light blue function (active only when the feedback is turned on – denoted by the gray shading) represents the explicit component ($x_{explicit}$), and the yellow and purple functions represent the fast (x_{fast}) and slow (x_{slow}) components of the implicit process, respectively.

1. Borg G a. V. Psychophysical bases of perceived exertion. *Med. Sci. Sports Exerc.* 1982;14:377.

2. Roemmich RT, Long AW, Bastian AJ. Seeing the Errors You Feel Enhances Locomotor Performance but Not Learning. *Curr. Biol.* 2016;26:2707–2716.

3. Smith MA, Ghazizadeh A, Shadmehr R. Interacting adaptive processes with different timescales underlie short-term motor learning. Ashe J, ed. *PLoS Biol.* 2006;4:e179.

4. Charalambous CC, Alcantara CC, French MA, et al. A single exercise bout and locomotor learning after stroke: physiological, behavioural, and computational outcomes: Effect of exercise on locomotor learning in chronic stroke survivors. *J. Physiol.* 2018;596:1999–2016.

5. Thoroughman KA, Shadmehr R. Learning of action through adaptive combination of motor primitives. *Nature*. 2000;407:742–747.

6. Wilson RC, Collins AG. Ten simple rules for the computational modeling of behavioral data. *eLife*. 2019;8:e49547.

7. Salvatier J, Wiecki TV, Fonnesbeck C. Probabilistic programming in Python using PyMC3. *PeerJ Comput. Sci.* 2016;2:e55.

8. Kim HE. bayes-toolbox: A Python package for Bayesian statistics. *J. Open Source Softw.* 2023;8:5526.

9. Kruschke J. *Doing Bayesian Data Analysis: A Tutorial with R, JAGS, and Stan*. Academic Press; 2014.

10. McElreath R. *Statistical Rethinking: A Bayesian Course with Examples in R and Stan*. CRC Press; 2016.
